# Physiological and transcriptomic analyses of yellow horn (*Xanthoceras sorbifolia*) provide important insights into salt and saline-alkali stress tolerance

**DOI:** 10.1371/journal.pone.0244365

**Published:** 2020-12-22

**Authors:** Juan Wang, Yunxiang Zhang, Xingrong Yan, Jinping Guo

**Affiliations:** 1 College of Forestry, Shanxi Agricultural University, Taigu, Shanxi, China; 2 Shanxi Key Laboratory of Functional Oil Tree Cultivation and Research, Taigu, Shanxi, China; ICAR-Indian Institute of Agricultural Biotechnology, INDIA

## Abstract

Yellow horn (*Xanthoceras sorbifolia*) is an oil-rich woody plant cultivated for bio-energy production in China. Soil saline-alkalization is a prominent agricultural-related environmental problem limiting plant growth and productivity. In this study, we performed comparative physiological and transcriptomic analyses to examine the mechanisms of *X*. *sorbifolia* seedling responding to salt and alkaline-salt stress. With the exception of chlorophyll content, physiological experiments revealed significant increases in all assessed indices in response to salt and saline-alkali treatments. Notably, compared with salt stress, we observed more pronounced changes in electrolyte leakage (EL) and malondialdehyde (MDA) levels in response to saline-alkali stress, which may contribute to the greater toxicity of saline-alkali soils. In total, 3,087 and 2,715 genes were differentially expressed in response to salt and saline-alkali treatments, respectively, among which carbon metabolism, biosynthesis of amino acids, starch and sucrose metabolism, and reactive oxygen species signaling networks were extensively enriched, and transcription factor families of bHLH, C2H2, bZIP, NAC, and ERF were transcriptionally activated. Moreover, relative to salt stress, saline-alkali stress activated more significant upregulation of genes related to H^+^ transport, indicating that regulation of intracellular pH may play an important role in coping with saline-alkali stress. These findings provide new insights for investigating the physiological changes and molecular mechanisms underlying the responses of *X*. *sorbifolia* to salt and saline-alkali stress.

## Introduction

Soil salinization and alkalization are important abiotic stress conditions that adversely affected growth and development processes in plants, such as seedling growth, tillering, metabolism and transcription [1–3]. Global human population expansion and inappropriate anthropogenic activities, such as overgrazing, deforestation, and improper soil and water management, are contributing to the salinization and alkalization of an ever-increasing expanse of land area [4]. Moreover, the formation and proportion of saline-alkali-affected land can be exacerbated by certain extreme weather/climatic events (i.e. droughts, floods, and heavy rainstorms) [5, 6]. Currently, approximately 950 million hectares of land are affected by salinization worldwide, and it is highly probable that the area adversely affected will undergo further expansion in the future [7–9]. To date, however, the majority of studies on saline-affected land have tended to focus on the neutral salt tolerance of plants, whereas comparatively less attention has been paid to plant tolerance to saline-alkali stress.

Salt stress can have a range of adverse effects on plants. Firstly, high salinity reduces the osmotic potential of the soil solution, and thereby inhibits water uptake by plants [10, 11]. Secondly, an excess accumulation of Cl^-^ and Na^+^ ions can induce specific ionic toxicities and nutrient imbalances [12–14]. Thirdly, salt stress may also promote an increase in the generation of reactive oxygen species (ROS), the excessive amounts of which can cause damage to intracellular components [15, 16]. Compared with salt stress per se, saline-alkali (NaHCO_3_ or Na_2_CO_3_) stress is associated with more severe damage to plants, owing to the combined adverse effects of salt stress, attributable high salt ion concentrations, and alkaline stress, which is related to high pH levels [17]. Furthermore, elevated pH levels may also lead to a deficiency in external protons and inhibit Na^+^/H^+^ antiport systems, thereby contributing to an accumulation of Na^+^ in plants [18, 19]. As counter-measures, plants have, however, evolved an array of salt/saline-alkali tolerance mechanisms, at both molecular and physiological levels, to facilitate continued growth and development, including antioxidant defence systems and osmotic adjustment [20]. In this regard, transcriptome analysis has emerged as a powerful approach for elucidating gene regulatory networks, and this has made a significant contribution to identifying numerous genes that are differentially expressed in response salt or saline-alkali stress in many cultivated plant species, including sugarcane [21], rice [22], alfalfa [23], grapevine [24], peach [25], wheat [26], and flax [27].

*Xanthoceras sorbifolia*, commonly known as yellow horn, is the only validated species in the genus *Xanthoceras* within the family Sapindaceae [28]. It is a woody deciduous shrub or small tree that is widely distributed in North China [29]. Yellow horn is considered an important bio-energy feedstock plant on account of its abundant content of seed kernel oil (55%–65%), which is rich in unsaturated fatty acids (85%–93%) [30, 31]. The plant is characterized by a strong adaptability to nutrient-poor, drought, cold, and saline-alkali conditions, and is typically found growing on marginal land [32]. Recently, *X*. *sorbifolia* has been receiving increasing attention, owing to its potential economic and biological importance, and several studies have presented transcriptomic data for this species, including those relating to oil accumulation [33, 34], fertilized ovule development [35], and abiotic stresses (i.e. salt, abscisic acid, and low temperature) [36, 37]. To date, however, no evidence has emerged to enable a comparative analysis of the mechanisms underlying the tolerance of *X*. *sorbifolia* to salt and saline-alkali stress.

Comprehensive and systematic studies that focus on the responses of plants to multiple abiotic stresses will contribute to distinguish the different processes associated with the adaptions of plants to each stress. In the present study, we accordingly adopted such an approach to examine changes in a selection of physiological indices and the activities of antioxidant enzymes in *X*. *sorbifolia* in response to treatment with NaCl or Na_2_CO_3_. Illumina sequencing technology was used to analyze the comparative transcriptome of *X*. *sorbifolia* seedlings subjected to salt and saline-alkali treatments to identify differentially expressed genes (DEGs) associated with stress tolerance. The aims of this study were to characterize the physiological changes in *X*. *sorbifolia* under salt and saline-alkali stress conditions, and to elucidate the molecular features related to salt and saline-alkali stress tolerance in this plant. These findings could contribute to determining the distinct physiological effects and gene reprogramming in response to these two stresses, and provide novel insights for further enhancing the adaptivity of *X*. *sorbifolia* to saline- and saline-alkali-contaminated environments.

## Materials and methods

### Plant materials and stress treatments

The *X*. *sorbifolia* superior tree (accession number: A099) from the breeding base of Shanxi Agricultural University (37°25N, 112°34E), with high oil content and a certain resistance to saline-alkali soil, was used in this study. Seeds were germinated at 25°C using the sand-hiding method. Following germination, seedlings were cultivated in a greenhouse at 23°C ± 2°C under a 14 h light/10 h dark photoperiod at a relative humidity of 60%-70%. The seedlings were irrigated regularly under natural conditions for a month. Healthy and uniform seedlings (height, ~30 cm) were transferred to tanks filled with Hoagland’s liquid medium for 1 week. All solutions were renewed at 2-day intervals. The seedlings were divided into three groups, with the plants in one group being maintained in Hoagland’s solution as controls. Seedlings in the remaining two groups were exposed to 150 mmol/L NaCl or 150 mmol/L Na_2_CO_3_ (pH 9.5) as salt and saline-alkali stress treatments, respectively. Previous studies have shown that solutions with a pH value higher than 10.0 may cause severe and rapid damage, whereas solutions with an insufficient alkalinity may make it difficult to study the responses to alkaline stress. Accordingly, a pH of 9.5 was deemed to be an appropriate pH value for examining the effects of alkaline stress [38]. At 0 (control), 4, 12, 24, and 48 h after commencing treatment, the leaves of seedlings were harvested for physiological analyses, and whole seedlings collected at 0 (control), 4, and 24 h were used as samples for transcriptome sequencing. Three biological replicates of each sample were taken for physiological analyses and transcriptome sequencing. We selected 24 h as the final time point for the purposes of transcriptome sequencing based on observations of the phenotypic changes occurring in *X*. *sorbifolia* seedlings in response to stress treatment compared with the control. All samples were immediately frozen in liquid nitrogen and stored at -80°C until further use.

### Physiological measurements

The electrolyte leakage (EL) value of leaves was measured using a digital conductivity meter as described in our previous study [37]. The contents of malondialdehyde (MDA) were measured using the thiobarbituric acid (TBA) colourimetric method with malondialdehyde assay kit (Solarbio, Beijing, China), and the contents of soluble sugars were detected using the thiobarbituric acid (TBA) colourimetric method with plant soluble sugar content assay kit (Solarbio, Beijing, China). Similarly, chlorophyll content was determined according to the protocol of a chlorophyll assay kit (Solarbio, Beijing, China). Chlorophyll was extracted using an anhydrous ethanol:acetone (1:2) solution, and the content was determined spectrophotometrically at 645 nm (for chlorophyll *a*) and 663 nm (for chlorophyll *b*). The soluble protein content of leaves was determined using Coomassie Brilliant Blue G-250 dye according to the Bradford method, with bovine serum albumin being used as the protein standard [39]. The content of MDA was expressed as micromoles of MDA per gramme fresh weight, whereas the contents of soluble sugars, soluble proteins, and chlorophyll were expressed in terms of milligrammes per gramme fresh weight.

### Enzyme activity assays

The activities of SOD, POD, CAT, and APX were determined according to the instructions provided with commercially available kits (Solarbio, Beijing, China). Fresh leaves (0.5 g) were ground to a fine powder in liquid nitrogen, and enzymes were extracted using the provided extraction buffers. The extracts thus obtained were centrifuged at 8,000 × *g* for 10 min at 4°C, and the resultant supernatants were used for further experiments. The activities of SOD, POD, CAT, and APX in the corresponding reaction mixtures were determined spectrophotometrically at 560, 470, 240, and 290 nm, respectively, and expressed in terms of units of enzyme activity per gramme fresh weight. All measurements were performed according to the instructions of the antioxidant enzyme assay kit.

Statistical analysis was conducted with an analysis of variance (ANOVA) and Student’s *t*-test using Statistical Package for the Social Sciences (SPSS) software (version 21.0; IBM SPSS Statistics, Armonk, USA).

### RNA extraction, library preparation, and RNA sequencing

Total RNA was extracted from plant samples using a TaKaRa MiniBEST Plant RNA Extraction Kit (TaKaRa, Dalian, China) according to the manufacturer’s protocol, and the integrity of the extracted RNA was monitored using 1% agarose gel electrophoresis. The purity and quality of RNA were assessed using a NanoDrop 2000C spectrophotometer (Thermo Fisher Scientific, Wilmington, USA) and an Agilent 2100 Bioanalyzer (Agilent Technologies, Santa Clara, USA), respectively. Sequencing libraries were constructed using an NEB Next^®^ Ultra^™^ RNA Library Prep Kit for Illumina^®^ (NEB, USA) following the manufacturer’s instructions. The prepared libraries were sequenced using an Illumina Hiseq 2500 platform (Illumina Inc., San Diego, CA, USA) of BIOMARKER (Beijing, China) and paired-end reads were generated. The raw sequence reads have been submitted to the Short Read Archive (SRA) of NCBI with BioProject accession number PRJNA608707 (Biosample: SAMN15763440–SAMN15763454).

### Transcriptome assembly and functional annotation

The *X*. *sorbifolia* transcriptome was assembled from 15 samples (three and 12 samples from control and stressed seedlings, respectively). Clean reads were obtained following the removal of reads containing adaptors and ploy-N sequences and low-quality reads from the raw data using the FastQC tool (http://www.bioinformatics.babraham.ac.uk/projects/fastqc/). The high-quality reads were assembled de novo into transcripts using Trinity [40]. Trinity combines reads with a certain length of overlap into longer contig sequences without gaps [41]. All assembled unigenes were annotated based on BLAST searches (E-value ≤ 1.0 × 10^−5^) of the Kyoto Encyclopaedia of Genes and Genomes (KEGG), Gene Ontology (GO), Clusters of Orthologous Groups (COG), eggnog, Swiss-prot, NR, and euKaryotic Orthologous Groups (KOG) databases. The software KOBAS2.0 [42] was used to obtain the KEGG orthology of unigene. The predicted amino acid sequences for each Unigene were aligned against the Pfam database [43] using HMMER [44] (E-value < 1.0 × 10^−10^) to acquire the annotation information of unigene. The sequenced reads were compared with the unigene library by Bowtie [45], and then the expression level was estimated with RSEM [46]. Gene expression levels were evaluated based on the fragments per kilobase of exon per million fragments mapped (FPKM).

Differential expression analysis was performed using the DESeq R package, with a false discovery rate-adjusted P value (FDR) < 0.05 and an absolute value of log2 FC ≥ 1 being used as the empirical parameters for identifying differentially expressed genes (DEGs). All DEGs were annotated using the aforementioned databases and the gene number for each GO term was calculated. The enrichment analysis of GO terms was conducted using the R package TopGO. KEGG enrichment of DEGs was performed using KOBAS software 2.0 [42]. Transcription factors (TFs) were predicted using the PlantTFDB database (http://planttfdb.gao-lab.org/index.php).

### Analysis of quantitative real-time PCR (qRT-PCR)

qRT-PCR experiments were performed using 20-μL reaction mixtures containing TB Green^®^ Premix Ex Taq^™^ II (TaKaRa, Dalian, China) in an ABI 7500 (Applied Biosystems, Carlsbad, USA), with *X*. *sorbifolia Actin* (c225347.graph_c0) being used as a reference gene and three independent biological replicates being analysed for each sample. Specific primers were designed based on sequencing results using Primer 5.0 software, the sequences of which are listed in S1 Table. The relative expression levels of amplified genes were determined using ABI 7500 sequence detection system software V2.3, and quantified by measuring cycle threshold (Ct) values, normalized relative to the expression of the Actin gene, using the 2^−ΔΔCt^ method.

## Results

### Physiological indices

**Fig 1:**
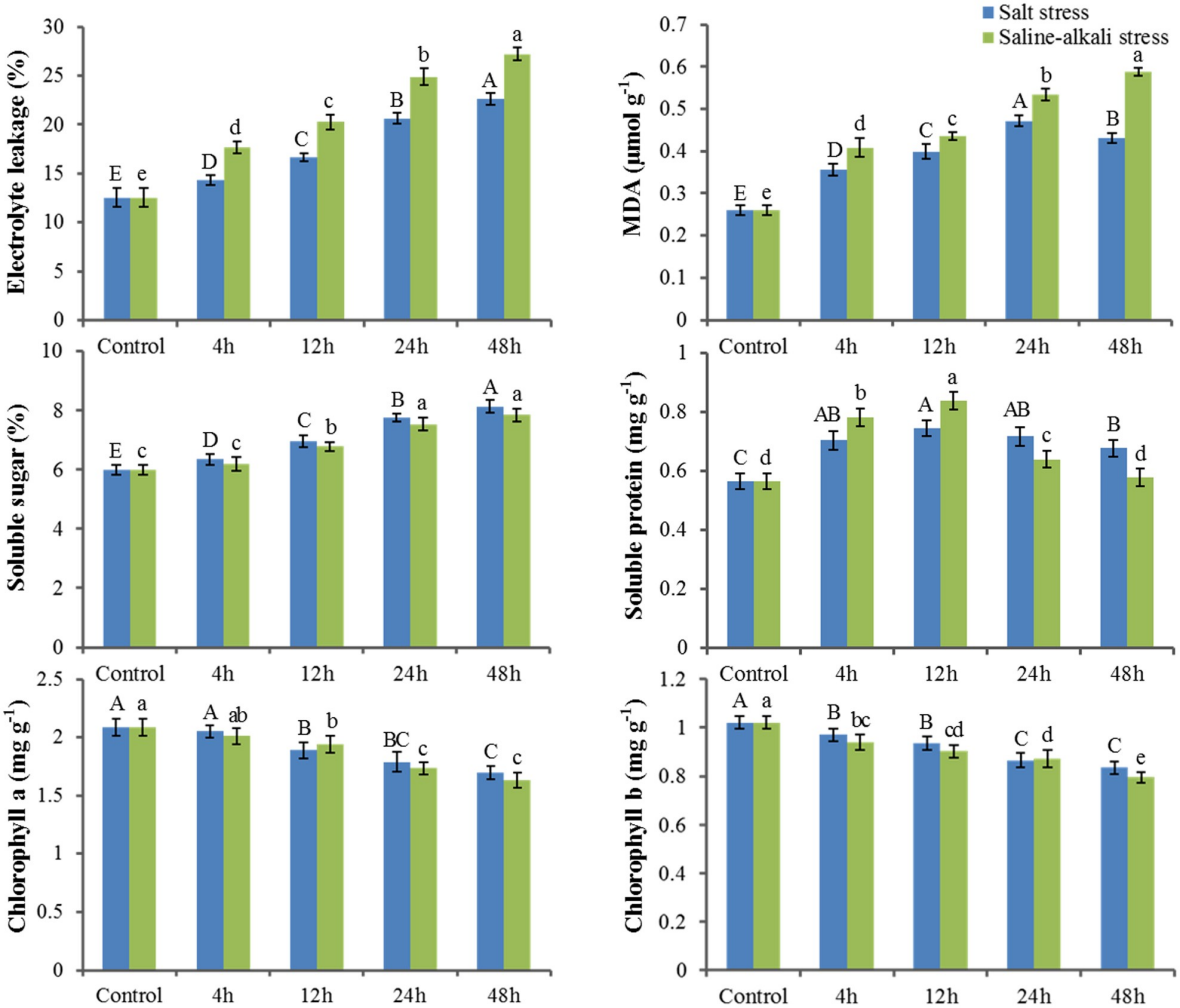
Changes in electrolyte leakage (EL), malondialdehyde (MDA), sugar, protein, and chlorophyll a/b in leaf tissues of *X*. *sorbifolia* under salt and saline-alkali stress. 4–48 h indicate different times of exposure to these two stresses. Error bars represent means ± SD (n = 3), and letters indicate significant statistical difference at p<0.05.

Compared with the control seedlings, salt and saline-alkali stress were found to promote increases in leaf EL and MDA, sugar, and protein contents, whereas leaf tissue chlorophyll content was observed to be reduced (Fig 1). The EL value increased almost linearly within 48 h of exposure to salt and saline-alkali stress, reaching levels 1.81- and 2.17-fold higher than the control, respectively. The content of MDA increased significantly in response to salt stress from 0 to 24 h, but subsequently underwent a gradual decline until the final measurement at 48 h. Under saline-alkali stress, MDA content increased rapidly within the initial 4 h, and thereafter gradually increased, peaking at 48 h. Notably, the levels of both EL and MDA in the saline-alkali treatment group were higher than those recorded for the control and salt stress groups. Leaf sugar content showed a slow increasing tendency with a prolongation of the time that seedlings had been exposed salt and saline-alkali stress. A similar tendency was observed for protein contents under the two stress treatments, with contents showing a marked increase at 12 h in treated leaves compared to with control leaves. Exposure to both salt and saline-alkali stress induced a continuous reduction in the contents of chlorophyll *a* and *b* until at least 48 h.

### Antioxidant enzyme activity

**Fig 2:**
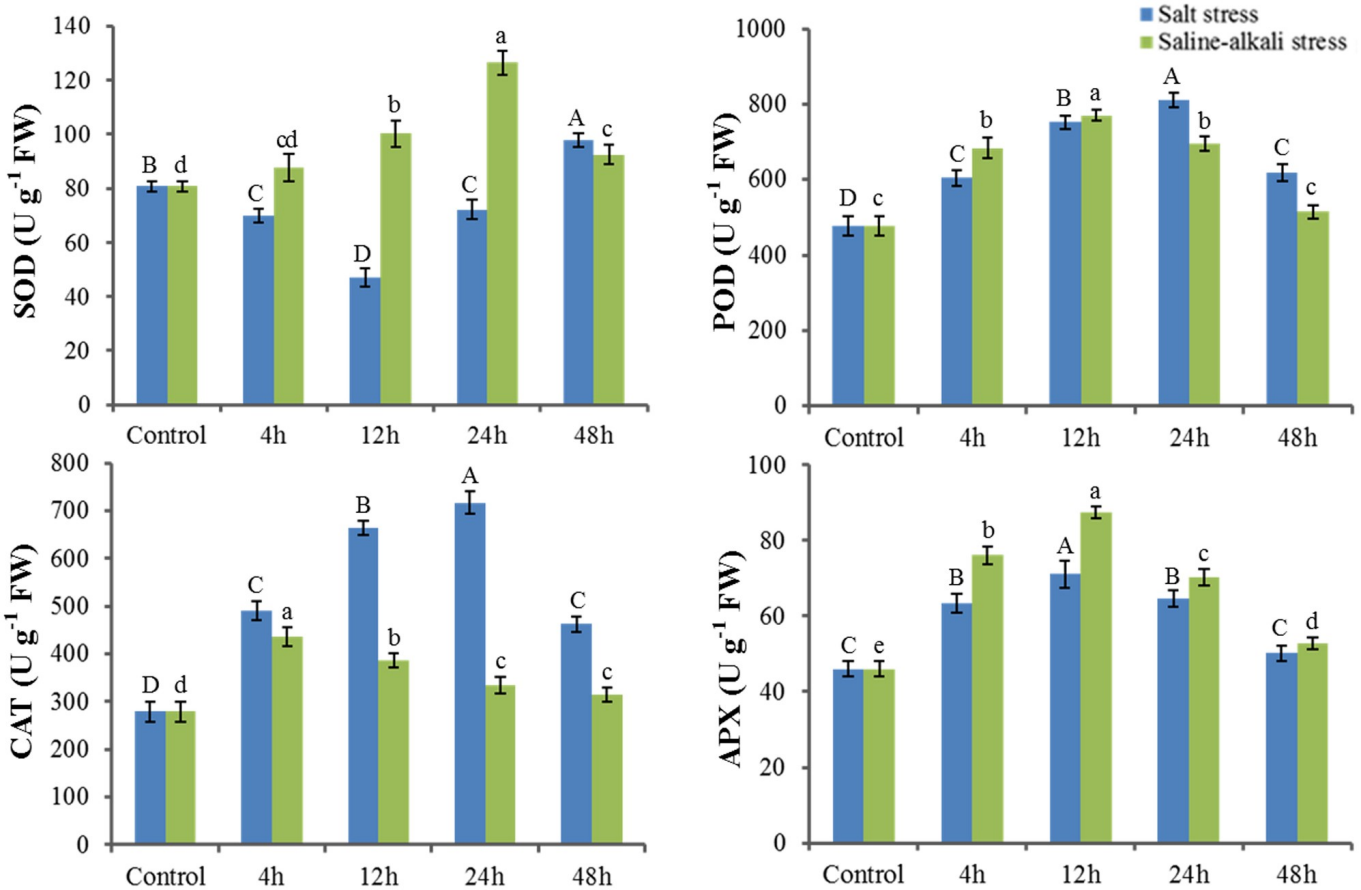
Changes in antioxidant enzyme activity in response to salt and saline-alkali stress. SOD, superoxide dismutase; POD, peroxidase; CAT, catalase; APX, ascorbate peroxidase. 4–48 h indicate different times of exposure to these two stresses. Error bars represent means ± SD (n = 3), and letters indicate significant statistical difference at p<0.05.

In response to salt stress, superoxide dismutase (SOD) activity showed an initial downward trend, followed by a subsequent increase, and at 48 h was significantly higher than that recorded in control seedlings. In contrast, under saline-alkali stress, SOD activity showed an initial slow increase within 4 h, then sharply increased until 24 h, but thereafter declined rapidly until 48 h. The peak value was, however, significantly higher than that recorded for the salt stress group (Fig 2). In response to both salt and saline-alkali stresses, the activities of peroxidase (POD), catalase (CAT), and ascorbate peroxidase (APX) appeared to follow a ‘rise and fall’ pattern as time progressed, and under both stresses, the activities were invariably higher than those recorded in control seedlings (Fig 2). POD activity peaked at 24 h and 12 h in response to salt and saline-alkali stress, respectively, whereas with respect to CAT, salt stress induced a greater increase in activity than did saline-alkali stress, particularly at 24 h. APX activity showed a similar tendency in response to each of the two stresses, although the change was observed to be more pronounced in response to saline-alkali stress.

### Transcriptome sequencing, assembly, and functional annotation

Following the removal of adaptors and low-quality sequences, the 15 cDNA libraries yielded approximately 90 Gb of clean reads. Q30 values were used to evaluate sequencing quality, and our findings that for each library, there were 93.07%–93.55% of bases scoring Q30 indicated that the RNA-Seq datasets were of high quality (S2 Table). Following assembly and data analysis, a total of 45,380 unigenes were obtained with an average length of 1,500 bp and N50 of 2,204 bp, including 24,313 unigenes with lengths exceeding 1 kb (S3 Table). Among these unigenes, 37,945 (83.62%) were annotated in at least one database. Notably, 36,388 (80.19%) unigenes showed significant hits using the NR database, whereas 22,341 (49.23%) unigenes showed significant matches to proteins in the Swiss-Prot database (Table 1).

**Table 1:** Functional annotation of the *X*. *sorbifolia* transcriptome.

Annotated Databases	Number of Unigenes	Percentage (%)
Annotated in COG	15,779	34.77
Annotated in GO	17,505	38.57
Annotated in KEGG	15,866	34.96
Annotated in KOG	22,018	48.52
Annotated in Pfam	29,164	64.27
Annotated in Swiss-Prot	22,341	49.23
Annotated in eggNOG	33,941	74.80
Annotated in NR	36,388	80.19
Annotated in at least one database	37,945	83.62
Total Unigenes	45,380	100

### Gene expression and identification of DEGs

**Fig 3:**
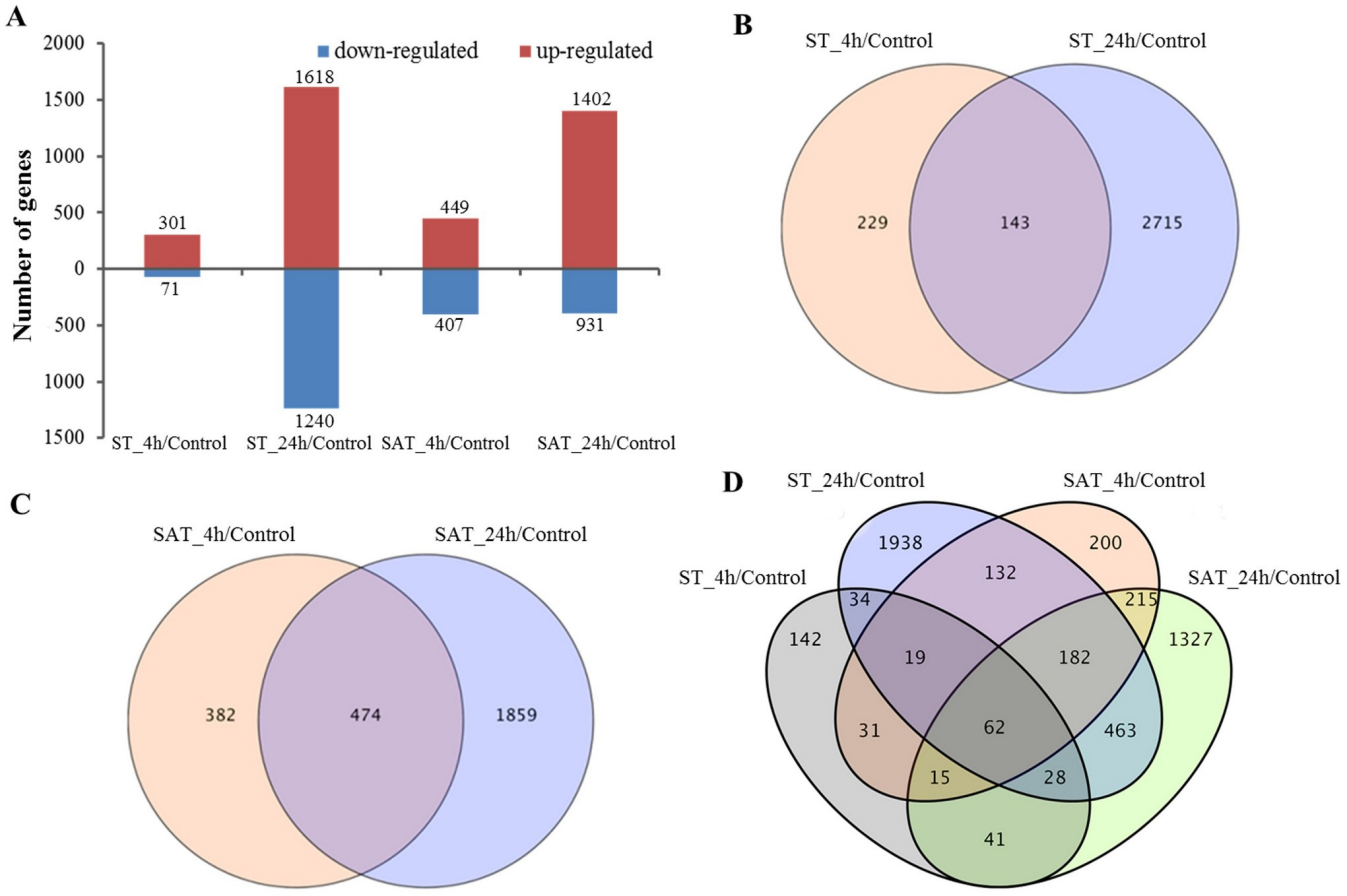
DEGs in the four pairwise comparisons of the control and stress treatments. (A) Bar chart showing the number of up- and downregulated genes in different comparisons. Venn diagram exposed the overlap of DEGs in two pairwise comparisons of salt stress (B), two pairwise comparisons of saline-alkali stress (C), and all four pairwise comparisons (D), respectively.

In order to identify DEGs, each of the treatment groups was compared with the control group. We accordingly identified 372 (301 up- and 71 downregulated) DEGs in the 4-h salt treatment group (ST_4h), 2858 (1,618 up- and 1,240 downregulated) DEGs in the 24-h salt treatment group (ST_24h), 856 (449 up- and 407 downregulated) DEGs in the 4-h saline-alkali treatment group (SAT_4h), and 2,333 (1,402 up- and 931 downregulated) DEGs in the 24-h saline-alkali treatment group (SAT_24h) (Fig 3A). These data indicate that the number of upregulated genes was greater than that of downregulated genes. Furthermore, we identified 62 DEGs showing response to both salt and saline-alkali stresses, 3,087 DEGs responding only to salt treatment, and 2,715 DEGs responding only to saline-alkali treatment (Fig 3B–3D). These stress-responsive genes detected at different sampling times under salt and saline-alkali stress were response- and time-specific, which may be related to the tolerance to different stresses.

**Fig 4:**
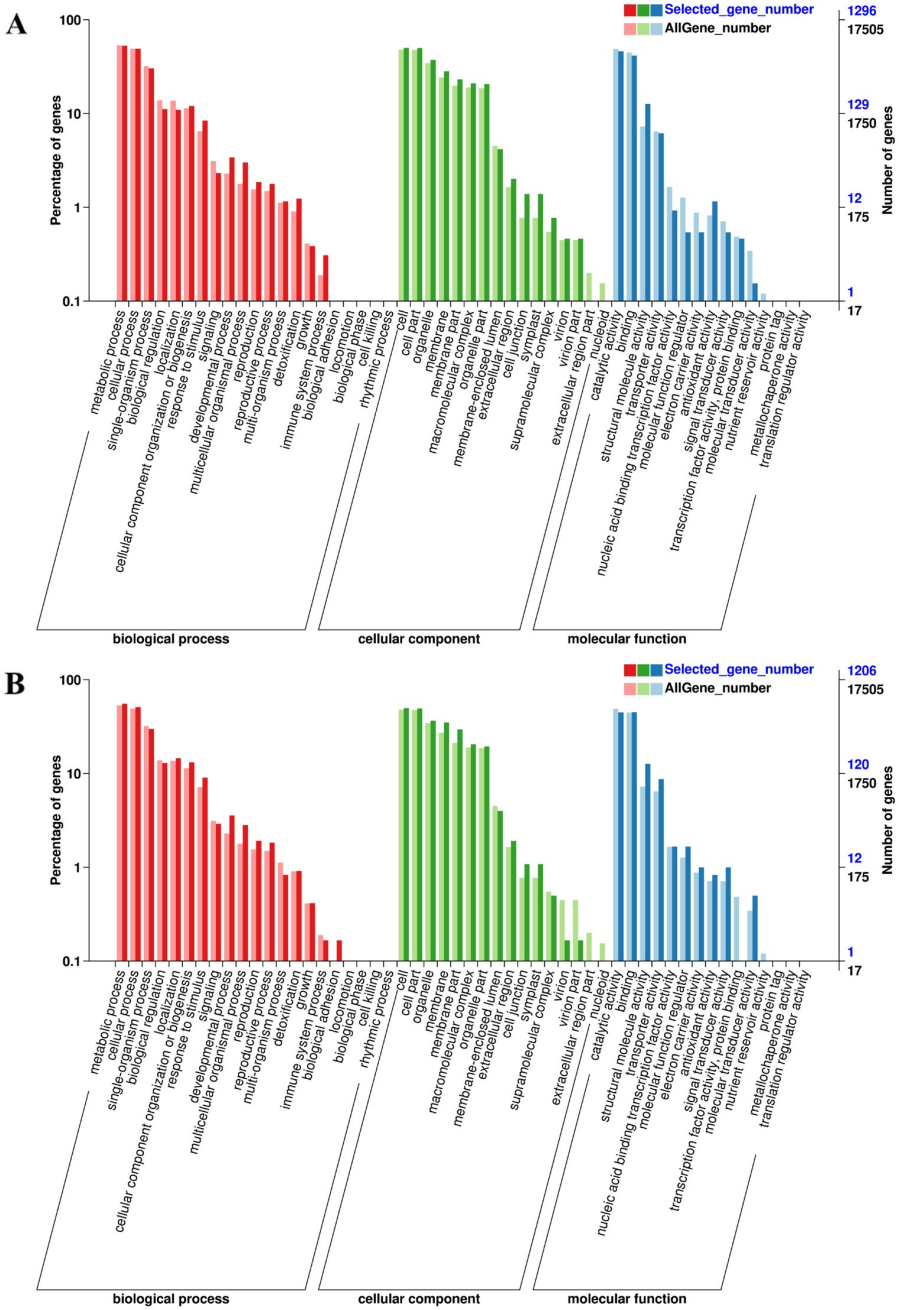
GO annotation of the DEGs, including the biological process, cell component and molecular function. (A) Enriched GO terms from salt-responsive DEGs. (B) Enriched GO terms from saline-alkali-responsive DEGs. Bold colours indicate the whole transcriptome gene complement, the light colours indicate DEGs.

For the purposes of GO analysis, we compared the GO terms for DEGs with those of the entire transcriptome gene complement. GO functional classification revealed that the DEGs could be divided into 51 functional groups, belonging to the three main GO domains: biological processes, cellular components, and molecular functions. For salt treatment, a total of 1296 (of 3,087) DEGs were associated with certain important GO terms, including response to stimulus, developmental process, detoxification, membrane, cell junction, symplast, structural molecule activity, and antioxidant activity (Fig 4A). For saline-alkali treatment, 1206 (of 2,715) DEGs were over-represented in the categories response to stimulus, developmental process, biological adhesion, membrane, cell junction, symplast, structural molecule activity, transporter activity, antioxidant activity, and signal transducer activity (Fig 4B).

**Fig 5:**
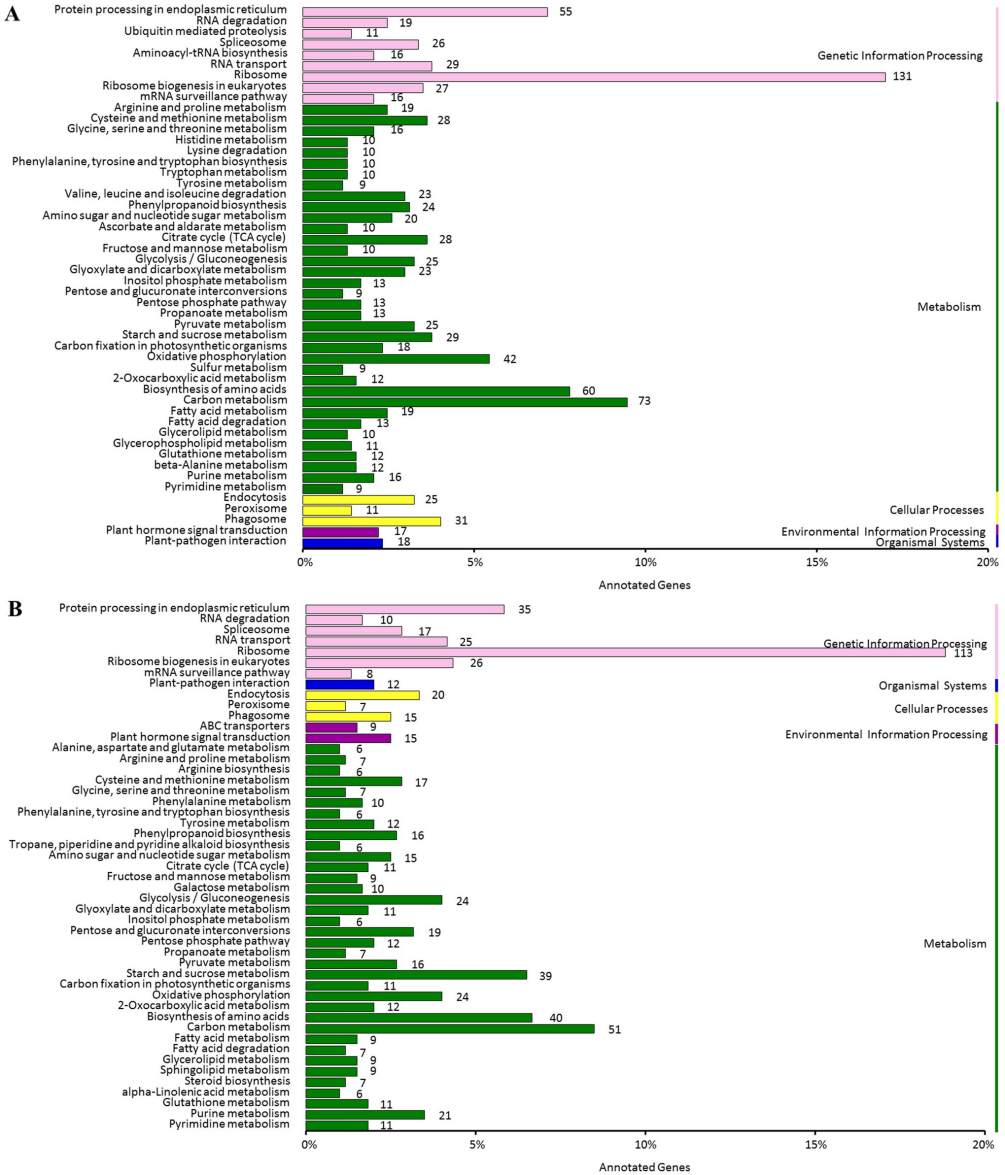
KEGG pathways enrichment of the DEGs. (A) The functional categories of the KEGG pathway enriched by salt-responsive DEGs are showed. (B) The functional categories of the KEGG pathway enriched by saline-alkali-responsive DEGs are showed.

Enrichment analysis of the DEGs based on KEGG annotation revealed that both salt-responsive and saline-alkali-responsive DGEs were significantly enriched with respect to ribosomes, carbon metabolism, biosynthesis of amino acids, starch and sucrose metabolism, and oxidative phosphorylation (Fig 5). Moreover, salt-responsive DGEs were significantly enriched in RNA transport, and the citrate cycle (TCA cycle), whereas saline-alkali-responsive DGEs were significantly enriched in glycolysis and gluconeogenesis and ABC transporters.

It should be noted that approximately 73 and 51 genes in plants subjected to salt and saline-alkali stress, respectively, were grouped into carbon metabolism pathways, among which several genes related to abiotic stress responses were identified (S4 Table). Two genes (c254724.graph_c0 and c260317.graph_c0) encoding transketolase (TKT) were upregulated under salt stress. The *TKT* gene (c257495.graph_c0) and phosphoglycerate kinase (PGK) gene (c260510.graph_c0) were expressed at higher levels in response to saline-alkali stress. However, two genes (c249246.graph_c1, c256367.graph_c0) encoding ribose-5-phosphateisomerase (RPI2) were downregulated in response to these two stresses.

The accumulation of osmolyte (i.e. sugar and amino acid) is an important feature for the protection and survival of plants in coping with abiotic stress. Some pathways related to sugar metabolism and amino acid metabolism were enriched with DEGs under salt and saline-alkali stress, such as biosynthesis of amino acids and starch and sucrose metabolism. At least 25 salt and 26 saline-alkali tolerance genes involved in the synthesis of osmolytes were detected in these pathways. (S5 Table), and most of them were upregulated. Four genes (c258340.graph_c0, c262255.graph_c1, c238817.graph_c0, and c238817.graph_c1) encoding trehalose phosphate synthases (TPS) showed significant upregulation under two stress conditions, whereas two *TPS* (c253905.graph_c0 and c236826.graph_c3) were upregulated only under salt stress. Several DEGs related to the metabolism of sucrose, glucose, proline, arginine, alanine, and betaine were also upregulated under salt stress. Moreover, DEGs for metabolism of sucrose, glucose, fructose, threonine, alanine, arginine, and cysteine were upregulated by saline-alkali treatment.

### Genes related to the ROS signaling network

**Fig 6:**
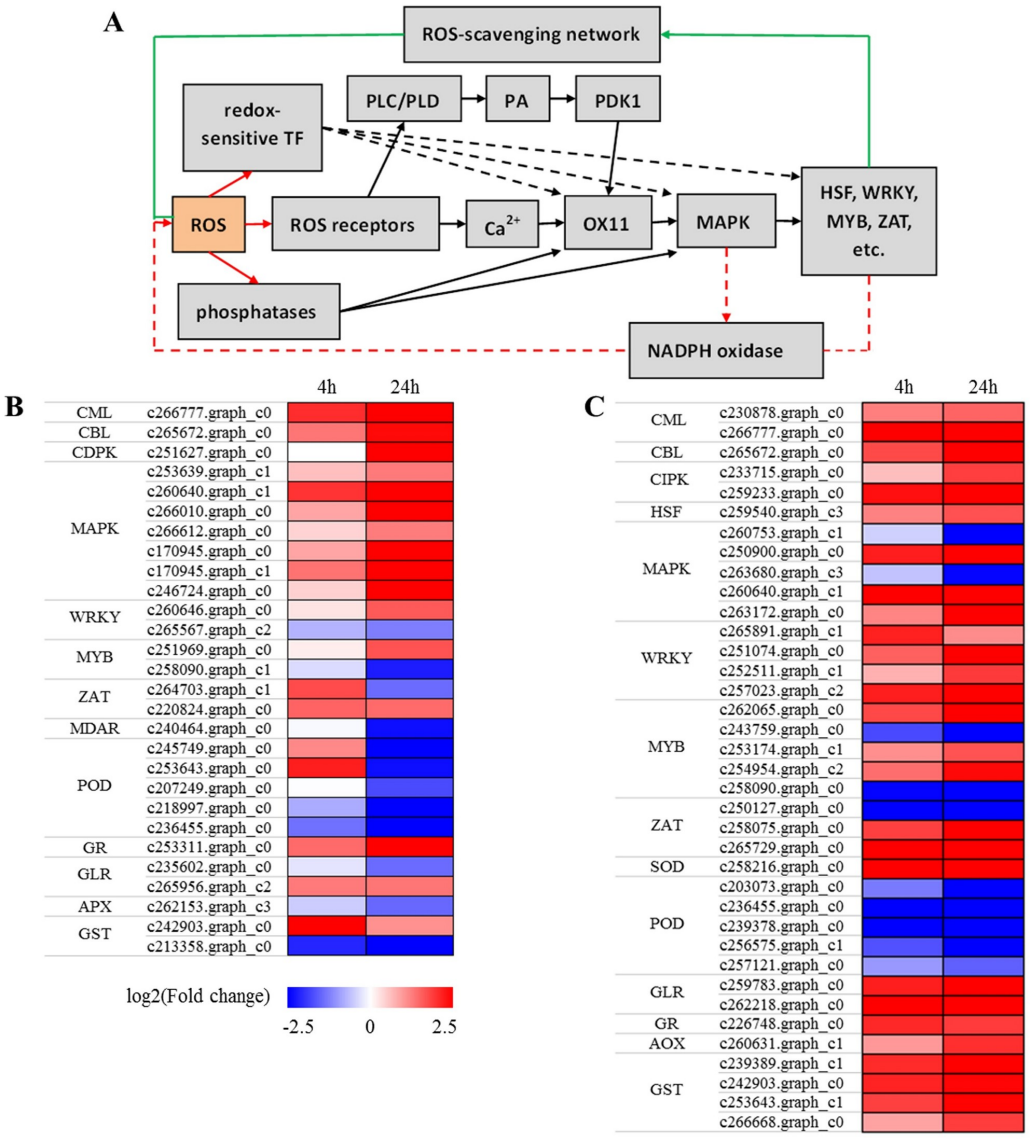
Heatmap of DEGs involved in ROS signaling network. (A) Overview of the ROS signal transduction pathway. (B) Expression patterns of salt tolerance DEGs are showed. (C) Expression patterns of saline-alkali tolerance DEGs are showed. CML, Calcium-binding protein; CBL, Calcineurin B-like protein; CIPK, CBL-interacting protein kinase; CDPK, Calcium-dependent protein kinase; HSF, Heat shock transcription factor; MAPK, Mitogen-activated protein kinase; WRKY, Wrky transcription factor; MYB, Myb transcription factor; ZAT, Zinc finger protein; MDAR, Monodehydroascorbate reductase; GLR, Glutaredoxin; GR, Glutathione reductase; AOX, Alternative oxidase; GST, Glutathione S-transferase.

ROS have been identified as serving a signaling function in plants, wherein they activate defence-related genes via redox-sensitive signaling pathways and transcription factors [47]. Schematic diagrams of the ROS-mediated signaling network are presented in Fig 6A [48]. In those seedlings subjected to salt treatment, we detected a total of 28 salt tolerance genes that are involved in the ROS signaling network, among which, 15 genes were upregulated at 4 h and 24 h of salt treatment. These were mainly distributed in the CML, CBL, CDPK, MAPK, and WRKY gene families. In contrast, six genes, which mainly encode ROS-scavenging enzymes, were downregulated in the two salt treatment groups (Fig 6B; S6 Table). In response to saline-alkali treatment, we detected 37 saline-alkali tolerance genes involved in the ROS signaling network, belonging to the CML, CBL, CIPK, HSF, MAPK, MYB, WRKY, ZAT families, or encoding ROS-scavenging enzymes. Compared with the control, with the exception of five POD genes that were all downregulated, most of the other gene families were upregulated in the two saline-alkali treatment groups (Fig 6C; S6 Table).

### Genes involved in H^+^ transport

**Fig 7:**
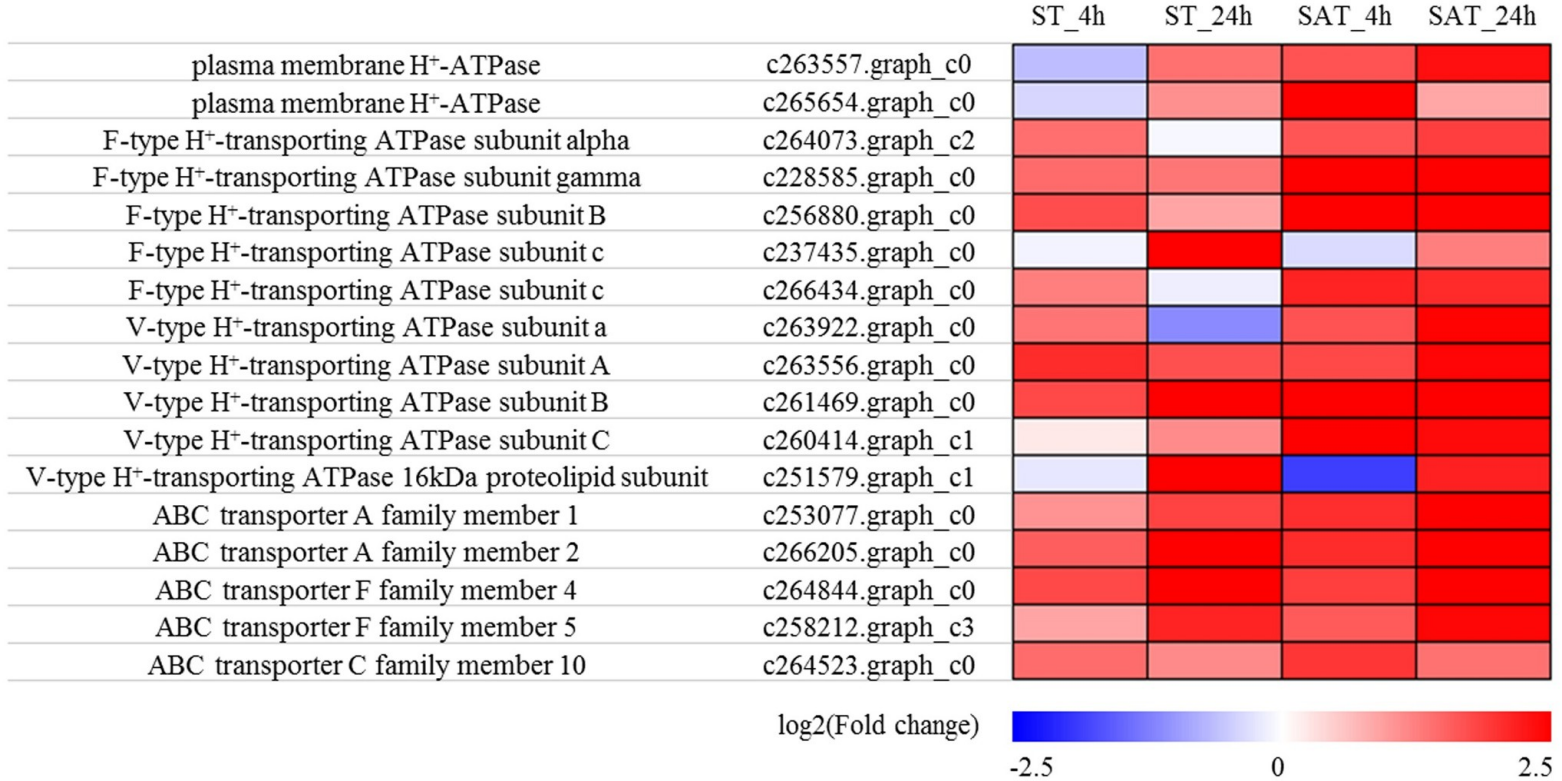
Expression patterns of DEGs involved in H^+^ transport in four pairwise comparisons of control and stress treatment groups.

We identified a total of 17 genes involved in H^+^ transport that were differentially expressed in response to the two stress treatments, among which there were two genes encoding plasma membrane H^+^-ATPase, five genes encoding F-type H^+^-transporting ATPase, five genes encoding V-type H^+^-transporting ATPase, and five genes encoding ABC transporters (Fig 7; S7 Table). The majority of these genes were upregulated in the four stress groups compared with the control group, and all genes showed a higher level of expression at 24 h in response to saline-alkali treatment, whereas lower expression levels were detected after 4 h salt treatment. Moreover, exposure to saline-alkali stress resulted in a more significant upregulation of these genes than exposure to salt stress.

### Dynamic expression of transcription factors

**Fig 8:**
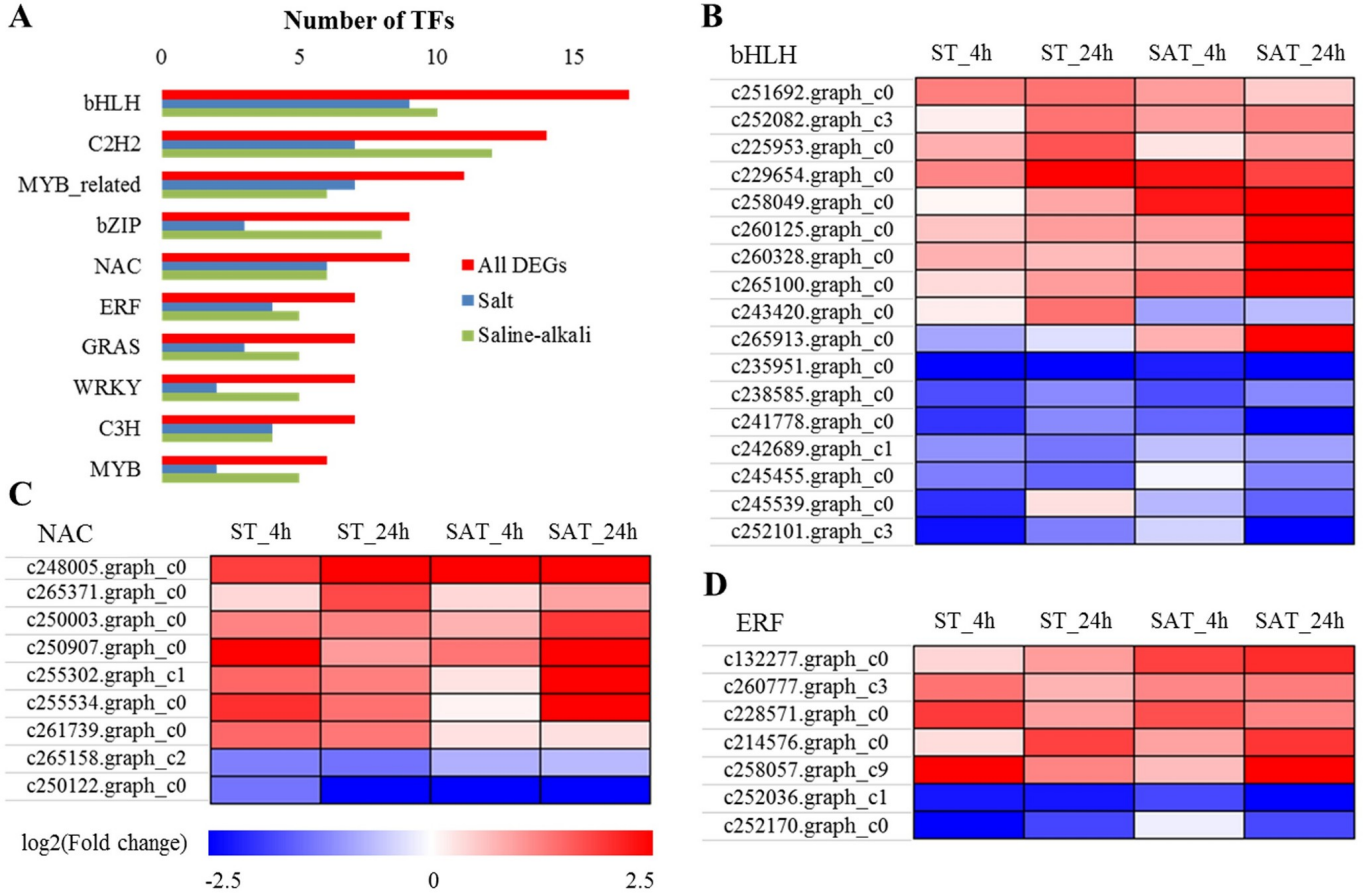
Classification and expression of transcription factors (TFs). (A) Distribution of DEGs into 10 major TF families that include more than six DEGs. (B) Heatmap of DEGs encoding the members of bHLH family. (C) Heatmap of DEGs encoding the members of NAC family. (D) Heatmap of DEGs encoding the members of ERF family.

Based on all 81 families of TFs predicted from the *Arabidopsis* TF database [49], we detected 34 families with at least one gene matched to the DEG dataset. 90 and 99 DEGs encoding TFs were identified in *X*. *sorbifolia* in response to salt and saline-alkali stress, respectively (S8 Table). Among these TF families, bHLH, C2H2, bZIP, NAC, and ERF families showed more active, and 10 TF families included more than six differentially expressed TFs (Fig 8A). A total 17 bHLHs were differentially expressed, nine of which were upregulated by saline-alkali treatments, and eight of which were upregulated by both salt and saline-alkali stress treatments (Fig 8B). The NAC family, with nine DEGs, with the exception of two genes (c265158.graph_c2 and c250122.graph_c0), seven genes were upregulated following exposure to these two stresses (Fig 8C). Furthermore, a total of nine DEGs belonging to the ERF family were identified, and seven of which were upregulated, whereas two genes (c252036.graph_c1 and c252170.graph_c0) were downregulated under salt and saline-alkali conditions (Fig 8D).

To further verify the accuracy of the RNA-Seq results, we selected 10 representative genes for qRT-PCR analysis, seven of which are involved in the ROS signaling network, two are associated with carbon metabolism, and one plays a role in H^+^ transport. Correlation analysis of the qRT-PCR and RNA-Seq results, based on scatter plotting of log2 (fold change) data, indicated that the qRT-PCR results were consistent with the sequencing data (Pearson coefficient r^2^ = 0.82, n = 40; S1 Fig).

## Discussion

Soil salinization and alkalinization often coincide in nature, inhibiting plant growth and leading to wilting or death [50]. Some salt-alkali soils have high salinity but low pH, while some have low salinity but high pH [51]. Therefore, neutral (NaCl or Na_2_SO_4_) and alkaline (NaHCO_3_ or Na_2_CO_3_) salts are usually defined as two distinct stresses. In this study, the physiological and RNA-Seq analyses were conducted on seedlings of *X*. *sorbifolia* exposed to salt (NaCl) and saline-alkali (Na_2_CO_3_) stress at different time point.

Environmental stresses can induce a range of physiological processes in plants, and in general, abiotic stresses have been found to activate oxidative responses and induce the production of ROS in plant cells [52]. ROS accumulation in turn enhances membrane lipid peroxidation due to a loss of cell membrane integrity, which negatively affects EL in response to abiotic stress [53–55]. Furthermore, the production of MDA, derived via lipid peroxidation, can exacerbate membrane damage [56]. Consequently, the extent of EL and amounts of MDA can reflect the degree of cell membrane damage incurred in response to different environmental stresses [57]. Indeed, MDA is commonly used as a marker of lipid peroxidation injury [58]. In the present study, we found that both salt and saline-alkali treatment resulted in increases in MDA concentrations and EL values in *X*. *sorbifolia*, indicating that the two stresses probably cause lipid peroxidation and disruption of the plasma membrane. Moreover, compared with the control seedlings, we found that the levels of MDA and EL were upregulated to a greater extent in response to saline-alkali stress than to salt stress, which we assume to be indicative of the greater harm caused by saline-alkali stress.

Soluble sugars, as effective osmoprotectants, can enhance the osmotic potential of plant cells to maintain ion homoeostasis [59]. Our study demonstrated that salt stress markedly increased the soluble sugar content of *X*. *sorbifolia* leaves, whereas the levels of these sugars were not significantly increased under conditions of saline-alkali stress. Interestingly, most genes involved in the metabolism of soluble sugars showed significant upregulation in our data (S5 Table). Protein synthesis has been considered a possible primary target of salt toxicity [60], and previous studies have shown that changes in soluble proteins in response to salinity tend to differ according to plant species and variety, plant developmental stage, and the duration and severity of salt exposure [61]. For example, a reduction in soluble protein content has been observed in tomato [62] and cabbage [59] under salt stress, whereas salt stress has been demonstrated to increase protein contents in maize [63] and *Zoysia macrostachya* [64]. In the current study, we found that the content of soluble proteins showed a ‘rise and fall’ tendency in response to the two imposed stresses. We suspect that this pattern could reflect an initial increase attributable to the expression of new stress-related proteins in *X*. *sorbifolia*, and a subsequent decrease in response to a marked decline in photosynthesis [65]. Notably, however, we found that the effects of saline-alkali treatment on protein content were more pronounced than those of salt treatment. Additionally, compared with the control, we detected gradual reductions in the contents of chlorophyll *a*/*b*, thereby indicating a decline in the rate of photosynthetic may be a common response to salt and saline-alkali stress in many plants [66–69].

SOD, POD, CAT, and APX are key protective enzymes that play vital roles in eliminating ROS and facilitating tolerance to abiotic stress. SOD functions by catalyzing the conversion of peroxide anions to H_2_O_2_ and O_2_, whereas POD, CAT, and APX catalyze the conversion of H_2_O_2_ to oxygen and water [70]. The physiological analyses conducted in the present study revealed certain differences in the antioxidant enzyme activities of *X*. *sorbifolia* seedlings exposed to salt and saline-alkali stress. The induction of the antioxidant system is dependent on the severity of the stress experienced by plants [71], and in this regard, our observations indicating that saline-alkali stress significantly induced SOD could imply that toxic superoxide might inflict greater damage in response to saline-alkali stress than when plants are exposed to salt stress. Our findings that the activities of POD, CAT, and APX appeared to show an initial increase, but then subsequently decreased, might reflect the fact that the initial slight stress stimulated the synthesis of oxidase enzyme, whereas later severe stress disrupted enzyme synthesis and perturbed enzyme degradation.

In plants, ROS are believed to play a key role in regulating signal transduction events during abiotic stress responses [72]. Plant cells are known to sense ROS through at least three different mechanisms, namely, unidentified receptor proteins, redox-sensitive transcription factors, and direct inhibition of phosphatases [48]. Downstream signaling events include calcium and phospholipid signaling pathways, and subsequent activation of serine/threonine protein kinase (OX11), MAPK cascades, NADPH oxidase, and transcription factors [48]. Furthermore, the generation of excess ROS can also trigger ROS-scavenging pathways and restricts the production of ROS in specific cellular locations or the entire cell. In the present study, the findings of our GO analysis revealed the significant enrichment of several ROS-related processes. A total of 28 and 37 differentially expressed genes related to the ROS signaling network were identified under salt and saline-alkali treatments, respectively. Previous studies have shown that numerous components of the ROS signaling network, including genes in the CML, CIPK, MAPK, MYB, WRKY, and HSF families, positively regulate the stress tolerance of plants [73–75]. Under salt and saline-alkali stresses, most genes in the CML, CBL, CDPK, MAPK, MYB, WRKY, and HSF families were upregulated. Additionally, we found that all almost of the identified genes encoding ROS-scavenging enzymes were downregulated to a greater extent in response to salt stress. In contrast, however, with the exception of POD genes, most genes encoding ROS-scavenging enzymes were upregulated following exposure to saline-alkali stress. We identified certain genes related to the ROS network that showed stress-specific expression, thereby indicating that in *X*. *sorbifolia*, different mechanisms regulate ROS homoeostasis in response to neutral salt and saline-alkali stress.

Saline-alkali stress affects plant nutrient absorption, growth, and photosynthesis via the combined effects of ion toxicity, osmotic stress, and high pH stress [13, 76, 77]. To counter the adverse effects of alkaline pH, plants can regulate intracellular pH through ion transport [19], and in this regard, several studies have shown that H^+^ transporter-related genes, such as plasma membrane H^+^-ATPase, F-type H^+^-transporting ATPase, and V-type H^+^-transporting ATPase, are positively regulated in response to salt or saline-alkali stress [78–80]. In the present study, we identified 17 H^+^ transporter-related DEGs, almost all of which were upregulated in response to salt and saline-alkali stress. Notably, a larger number of H^+^ transporter-related genes were upregulated under saline-alkali conditions, and the expression level of the upregulated genes tended to be higher than that of those genes upregulated in response to salt stress. These results are consistent with those reported by Zhang et al. [81], and we accordingly speculate that the higher expression of these genes may make an important contribution to maintaining intracellular ion balance and counteracting the negative effects of high pH associated with saline-alkali stress.

TFs are regulatory components in transcriptional networks and are involved in various processes, including plant development, hormone signaling, and stress response [82, 83]. In the present study, a number of TFs were differentially expressed under salt and saline-alkali stress, most of which belonged to bHLH, MYB, C2H2, bZIP, NAC, ERF, GRAS, WRKY, and C3H families, which is consistent with findings in certain plants [25, 67, 84]. It has been confirmed that members of the transcription factor family such as bHLH, NAC, ERF, MYB, and WRKY are involved in plant response to abiotic stress [85, 86]. A fraction of TFs, including 8 bHLHs, 7 NACs, and 5 ERFs, were upregulated under both salt and saline-alkali stress, implying their important roles in the regulation of *X*. *sorbifolia* responses to salt and saline-alkali stress.

In summary, this study is the first to report a comprehensive physiological and transcriptomic analysis of the responses of *X*. *sorbifolia* to salt and saline-alkali stress. We observed certain physiological changes in *X*. *sorbifolia* seedlings that had been subjected to salt and saline-alkali stress treatments, including ROS accumulation, membrane lipid peroxidation, and the reduction of chlorophyll content. Furthermore, on the basis of transcriptomic datasets, we identified a large amount of genes and pathways related to stress responses. These data enabled us to characterize the common and contrasting features of salt and saline-alkali stress tolerance in *X*. *sorbifolia*, will contribute to distinguish the response mechanisms of this species to these two stresses. Also, for the future, systemic investigation of candidate gene will be required to extend our results.

## Supporting information

S1 Fig The expression correlation of 10 selected genes between qRT-PCR and RNA-Seq data. (TIF)

S1 Table Primers used in qRT-PCR analysis. (XLS)

S2 Table Overview of the transcriptome sequencing data. (XLS)

S3 Table Statistics of the unigene assembly results. (XLS)

S4 Table Salt and saline-alkali tolerance genes related to carbon metabolism. (XLS)

S5 Table Salt and saline-alkali tolerance genes for osmolytes. (XLS)

S6 Table Salt and saline-alkali tolerance genes related to ROS signaling network. (XLS)

S7 Table Salt and saline-alkali tolerance genes related to H^+^ transport. (XLS)

S8 Table DEGs encoding transcription factor families. (XLS)
